# Insights into the Incidence, Course, and Management of Lithium-Induced Hypothyroidism in Real-World Psychiatric Practice in Italy

**DOI:** 10.3390/ph17111425

**Published:** 2024-10-24

**Authors:** Simone Pardossi, Mario Pinzi, Matteo Cattolico, Maria Beatrice Rescalli, Lorenzo Nicchi, Benedetta Tuci, Elisa Mariantoni, Alessandro Cuomo

**Affiliations:** Department of Molecular Medicine, School of Medicine, University of Siena, 53100 Siena, Italy; s.pardossi@student.unisi.it (S.P.); mario.pinzi@student.unisi.it (M.P.); m.cattolico1@student.unisi.it (M.C.); m.rescalli@student.unisi.it (M.B.R.); l.nicchi@student.unisi.it (L.N.); b.tuci@student.unisi.it (B.T.); e.mariantoni@student.unisi.it (E.M.)

**Keywords:** lithium, hypothyroidism, bipolar disorder

## Abstract

**Background**: Lithium is a cornerstone in the treatment of bipolar disorder (BD). However, lithium use requires careful monitoring of thyroid function due to associated dysfunctions. The aim of our real-world study is to retrospectively evaluate the impact of lithium on thyroid function and how these thyroid alterations can be measured and managed. **Methods**: A retrospective observational study was performed on 150 patients with BD who started lithium treatment at the University Hospital of Siena. Thyroid function was assessed at baseline and after the introduction of lithium by measuring TSH, T3, and T4 levels at baseline and after 3, 6, 9, and 12 months, during which changes in psychiatric symptoms were also evaluated using specific psychometric scales. **Results**: Significant increases in TSH levels were observed at 3 and 6 months, while T3 and T4 levels decreased significantly at 3 months. Transient thyroid dysfunction occurred in 36.7% of patients, but normalized without the discontinuation of lithium or need for thyroid replacement therapy in most cases; however, replacement therapy was initiated in 8.7% of patients. There were no significant differences in treatment response between patients with and without thyroid abnormalities, as the abnormalities were transient or resolved. **Conclusions:** In our sample, lithium induced some cases of hypothyroidism, which, being transient or corrected with replacement therapy, did not interfere with symptomatic improvement. These findings underscore the necessity for continuous thyroid function monitoring during lithium therapy. Clinicians should be prepared to initiate thyroid replacement therapy, when necessary, as timely management can prevent the interruption of lithium treatment and ensure ongoing symptomatic improvement in BD patients. Future studies could include larger and more diverse populations to validate these findings further, extending the follow-up period beyond 12 months to better observe long-term thyroid function trends and management outcomes.

## 1. Introduction

Bipolar disorder (BD) is a significant mental health condition affecting over 1% of the global population [1]. This disorder presents with distinct clinical features, including manic and depressive episodes, and sometimes mixed features that exhibit symptoms of opposite polarities within the same episode [1,2,3,4]. The chronicity and complexity of BD contribute to its underdiagnosis and significant clinical and socioeconomic burden [5,6]. Studies show that many patients with BD experience symptoms approximately 50% of the time over follow-up periods exceeding ten years [6]. Comorbidity further complicates the diagnosis and treatment of BD [7,8].

Pharmacological treatments for BD include mood stabilizers like lithium, antipsychotics, and anticonvulsants [4,9]. Lithium remains a gold standard treatment for BD, with robust evidence supporting its mood-stabilizing effects, which extend to both manic and depressive episodes, as well as its anti-suicidal efficacy [10,11]. The BALANCE study confirmed lithium’s efficacy in preventing relapse and reducing suicidality in BD patients [12]. A comprehensive systematic review by Fountoulakis et al. [10] reviewed the evidence concerning lithium’s efficacy in various phases and clinical facets of BD, supporting lithium’s usefulness across a broad spectrum of clinical issues in BD, particularly in treating acute mania, including cases with psychotic symptoms. The review also supports the use of lithium in combination therapies, such as with olanzapine and quetiapine, in conditions such as mixed states [10].

Lithium’s mechanism of action in treating BD involves inhibiting glycogen synthase kinase-3β (GSK-3β), impacting neuronal plasticity, neuroprotection, and mood regulation [13,14]. It modulates neurotrophic factors, neurotransmitters, oxidative metabolism, apoptosis, and second messenger systems, contributing to mood stabilization [13]. Lithium protects neurons from excitotoxicity [15] and affects intracellular calcium homeostasis [16]. It reduces tau phosphorylation, potentially benefiting neurodegenerative conditions [17], and normalizes intracellular sodium levels [18]. Lithium influences gene transcription through the cyclic AMP response element-binding protein (CREB) [19] and may increase brain structure volumes involved in emotional regulation [20]. It also enhances neuroprotective proteins like BDNF and Bcl-2, reducing oxidative stress and inflammation [20].

Lithium is concentrated in the thyroid at levels three to four times higher than in the plasma [21]. This drug reduces the uptake of radioiodine in the thyroid of rats and other species, suggesting that it may compete for iodide transport [22]. One of the most significant effects of lithium on the thyroid is the inhibition of thyroid hormone release, leading to the development of goiter and hypothyroidism [21]. This inhibitory effect involves alterations in tubulin polymerization and the inhibition of TSH action on cyclic AMP [21]. Additionally, lithium can affect the activity of deiodinases, the enzymes responsible for the conversion of thyroid hormones, further contributing to thyroid functional alterations [21,23]. Lithium increases thyroid autoimmunity (as evidenced by positive thyroid peroxidase antibodies) if present before therapy but does not cause de novo synthesis of antibodies in animals or humans [21,24]. Lithium might be associated with a range of thyroid dysfunctions, including hypothyroidism, hyperthyroidism, and thyroiditis. The frequency of these dysfunctions varies, with hypothyroidism being the most common, affecting 6–52% of patients on lithium therapy [25]. Thyroiditis and hyperthyroidism are less common but still significant, with a prevalence of about 0.5–10% for hyperthyroidism [26]. Persistent hypothyroidism is more common than transient forms, with women, particularly those with pre-existing thyroid autoimmunity, a family history of thyroid disease, or a history of postpartum thyroiditis, being at higher risk [27]. Additionally, factors such as the duration and cumulative dose of lithium therapy have been implicated as risk factors, where prolonged exposure increases the likelihood of developing thyroid dysfunction [25]. Age also plays a role, with older adults being more susceptible to lithium-induced thyroid dysfunction [26]. Studies suggest that about 20–30% of patients on long-term lithium therapy may require thyroid hormone replacement due to the development of clinically significant hypothyroidism [27].

The impact of lithium on thyroid function has also been studied in the context of ultrasonographic changes in the thyroid gland, with findings indicating alterations in thyroid volume and morphology in lithium-treated individuals [28,29]. Moreover, the potential reversibility of lithium-associated hypothyroidism upon the discontinuation of lithium treatment underscores the dynamic nature of lithium’s effects on thyroid function [30].

Thyroid function monitoring should begin before starting lithium treatment and continue with checks every 3–6 months during the first year of therapy, and then every 6–12 months thereafter. The minimum required test is the thyroid-stimulating hormone (TSH) level, with some experts also recommending the measurement of peripheral thyroid hormones (T3 and T4) and antithyroid antibodies [31].

In a 2006 study, it was found that 38% of patients with bipolar disorder treated primarily with lithium developed abnormalities in TSH and/or free thyroxine index (FTI) values. Additionally, these patients spent significantly longer in the acute phase of treatment and showed higher average scores on the Hamilton Scale for Depression during the maintenance phase. A significant correlation was found between the duration of lithium treatment and the development of abnormal thyroid values [32].

The aim of this study was to explore the potential effects of lithium on thyroid function in a real-world cohort of bipolar disorder patients, focusing on how these changes can be identified and managed over time. Specifically, this research seeks to verify and quantify the effect of lithium therapy on the development of hypothyroidism in a population of BD patients undergoing treatment in Italy

## 2. Results

### 2.1. Participants

One hundred and fifty patients (women: 51.3%, men: 48.7%, median age 47 years, IQR: 28–58 years) were included in the study. In this group, a thorough collection of medical histories was conducted, and it was determined that none of the patients had any prior conditions associated with thyroid dysfunction. Additionally, it was confirmed that none of the patients were taking any medications known to affect thyroid function Patients’ demographic and clinical characteristics are shown in Supplementary Materials, Table S1. All subjects received lithium at variable dosages, adjusted on a clinical basis throughout the 12-month observation period. Patients were allowed to receive thyroid substitution therapy as deemed necessary by an endocrinologist. The need for substitution therapy increased slightly over time. At the baseline (0 and 3 months), none of the 150 patients required substitution therapy. However, by 6 months, 1 patient (0.7%) had initiated therapy. This number increased to 2 patients (1.3%) at 9 months, and by the end of the 12-month period, 5 patients (3.3%) were receiving substitution therapy.

Other psychopharmacological treatments were also allowed. Eighty percent of patients were concomitantly treated with lithium and SSRIs; concurrent treatments with second-generation antipsychotics (76.0%), mood stabilizers (64.7%), third-generation antipsychotics (53.3%), and benzodiazepines (49.3%) were also recorded.

### 2.2. Thyroid Functionality Variations

**Figure 1:**
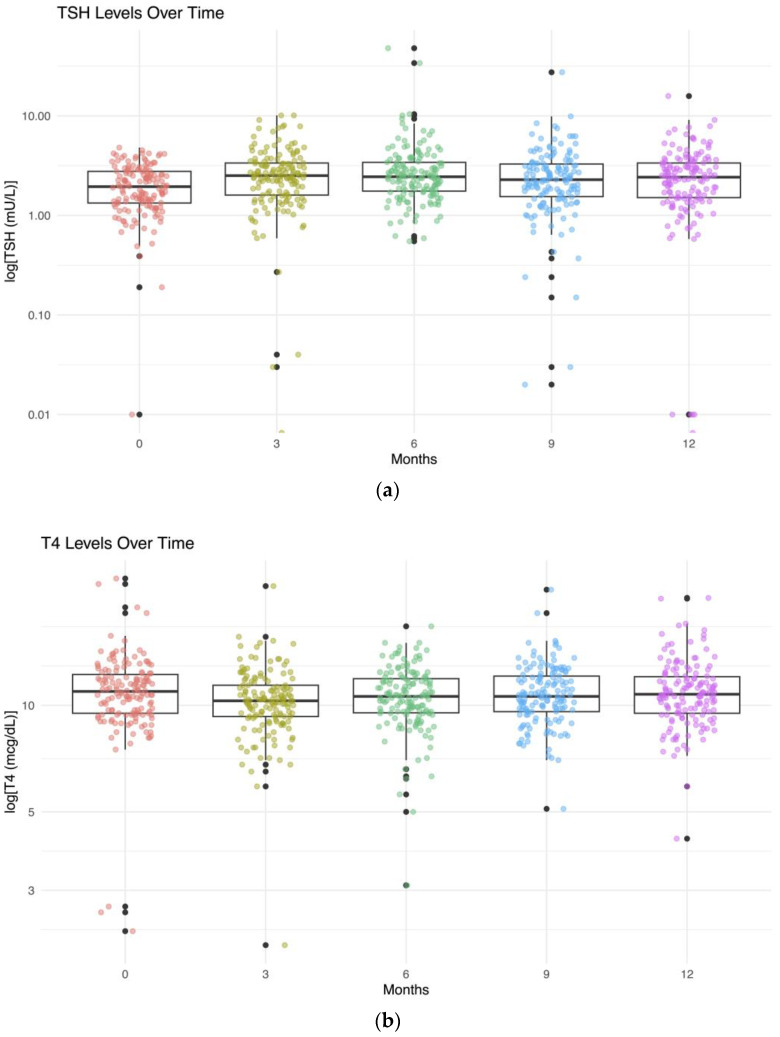
(**a**,**b**) TSH levels over time in patients undergoing lithium therapy. This boxplot illustrates the distribution of TSH (in mU/L) and T4 (in mcg/dL) levels in log scale at baseline (month 0) and at subsequent time points (3, 6, 9, and 12 months) during lithium treatment. Each colored dot represents an individual patient’s TSH level at a specific time point, with colors corresponding to different months. The boxes indicate the interquartile range (IQR), with the horizontal line inside each box representing the median TSH level. Whiskers extend to 1.5 times the IQR, and individual points beyond the whiskers represent outliers. The plot highlights the trend of TSH levels over time, showing that while most patients maintain TSH levels within a typical range, a few exhibit elevated levels, particularly at the 6-month mark.

At baseline (month 0), the mean TSH level was 2.09 mU/L (SD = 1.01), with values ranging from 0.01 to 4.8 mU/L and a median of 1.94 mU/L. At month 3, the mean was 2.86 mU/L (SD = 1.86), 3.35 mU/L (SD = 4.78) at month 6, 2.80 mU/L (SD = 2.62) at month 9, and 2.73 mU/L (SD = 1.96) at month 12 (Figure 1a,b).

For T3 levels, the mean at baseline was 3.08 nmol/L (SD = 1.05), with a range of 1.6 to 9.5 nmol/L and a median of 2.9 nmol/L. At month 3, the mean T3 level was 2.87 nmol/L (SD = 0.724), and by month 12, it was 3.03 nmol/L (SD = 0.932).

T4 levels had a baseline mean of 11.1 pmol/L (SD = 2.60), 10.4 pmol/L (SD = 2.19) at month 3, and 11.0 pmol/L (SD = 2.36) by month 12.

Lithium levels were 0 mmol/L at baseline, had a mean of 0.540 mmol/L (SD = 0.215) at month 3, 0.558 mmol/L (SD = 0.264), 0.544 (SD = 0.220), and 0.591 mmol/L (SD = 0.22) at month 12. The mean lithium doses were 0 mg at baseline, 682 mg (SD = 140) at month 3, 692 mg (SD = 150) at month 6, 692 mg (SD = 140) at month 9, and 710 mg (SD = 146) at month 12 (Supplementary Materials, Table S2).

**Figure 2:**
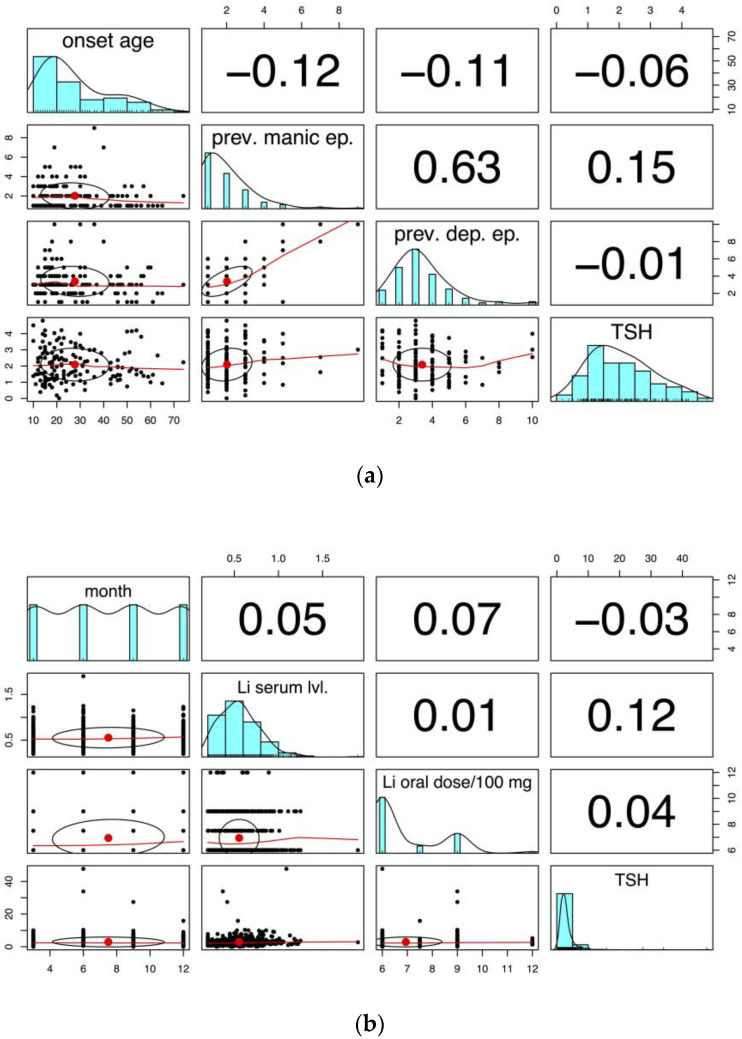
(**a**) Onset age, number of previous manic and depressive episodes, and their relationships with TSH levels. The diagonal represents histograms for each variable, the scatterplots represent pairwise relationships, and the correlation coefficients are displayed in the upper triangle. (**b**) Time (in month) lithium serum levels and oral dose and their relationships with TSH levels. The diagonal represents histograms for each variable, the scatterplots represent pairwise relationships, and the correlation coefficients are displayed in the upper triangle.

No strong linear correlations were observed between the TSH levels and any of the variables, as reflected by the low correlation coefficients (Figure 2a,b).

Patients were classified into thyroid status categories based on TSH and T4 blood levels using the following criteria: TSH > 10 and T4 < 9 was classified as hypothyroidism, TSH > 10 as subclinical hypothyroidism, and TSH ≤ 10 as normal. During the study period, transient abnormal thyroid levels were observed in the patient population. At baseline, all 150 patients were either normal (142) or subclinical (8), with no cases of hypothyroidism. By month 3, there were 122 normal, 27 subclinical, and 1 hypothyroid patient. At month 6, there were 123 normal, 24 subclinical, and 3 hypothyroid patients. At month 9, the distribution was 125 normal, 24 subclinical, and 1 hypothyroid patient, and by month 12, there were 127 normal, 21 subclinical, and 2 hypothyroid patients. Descriptive Statistics by Substitution Therapy Status are shown in Supplementary Materials, Table S3, Figure S1. Although several patients experienced abnormal thyroid levels during the study, only five required replacement therapy due to elevated TSH levels accompanied by symptoms such as weight gain, cold intolerance, and weakness.

After considering the substitution therapy as indicating hypothyroidism, the classification changed as follows: at month 3, 1 patient was categorized as hypothyroid; at month 6, there were 4 hypothyroid patients; at month 9, the number decreased to 3 hypothyroid patients; and by month 12, 7 patients were classified as hypothyroid, with 126 patients remaining normal and 17 subclinical.

### 2.3. Clinical Results

All the patients in our sample were in a predominantly depressive phase (MADRS: median = 28, 25% = 26, 50% = 28, 75% = 30). The MADRS and MRS scores, however, exhibited a significant linear decline beginning from the third month (the first evaluation time point considered after the baseline), with 98% of the patients achieving complete remission (total MADRS score ≤ 12 points) at nine months. More than 75% of patients responded to treatment in the first six months (MADRS ≥ 50% improvement from baseline). At month three, 148 out of 150 patients showed a CGI response, with 80 patients (53.3%) achieving minimal improvement (CGI = 3), 68 patients (45.3%) achieving moderate improvement (CGI = 2), and no patients achieving full remission (CGI = 1). No differences in treatment response were observed in patients with and without thyroid abnormalities.

### 2.4. Main Results

The final model, resulting from the selection process described, includes lithium serum levels and their interaction with time as the main variables of interest, along with additional covariates used to adjust for potential confounding factors such as the baseline TSH, gender, treatment with gabapentin or pregabalin, the use of other antidepressants (not SSRIs or SNRIs), and age at onset.

The results indicate that lithium serum levels significantly impact the progression of thyroid dysfunction, with an odds ratio (OR) of 1.29 (95% CI: 1.12 to 1.48) for each 0.1 mEq/L increase in serum lithium levels. This suggests that for every 0.1 mEq/L increase in lithium serum levels, the odds of progressing to a more severe category of thyroid dysfunction increase by about 29%.

The interaction between lithium serum levels and time shows an OR of 0.98 (95% CI: 0.96 to 1.00) per month, suggesting that the impact of lithium on thyroid dysfunction decreases slightly over time, indicating a potential adaptation or stabilization of thyroid function with prolonged lithium exposure.

The random effects component of the model, represented by patient-specific intercepts (variance = 1.899, standard deviation = 1.378), indicates considerable between-subject variability in baseline thyroid function, highlighting the importance of accounting for individual differences in clinical analyses.

The final model, using lithium oral daily dose as the main exposure variable, resulted from the selection process described earlier and includes similar confounding variables as the previous model: the baseline TSH, gender (with male as the reference), treatment with gabapentin or pregabalin, the use of other antidepressants (not SSRIs or SNRIs), and age at onset.

**Figure 3:**
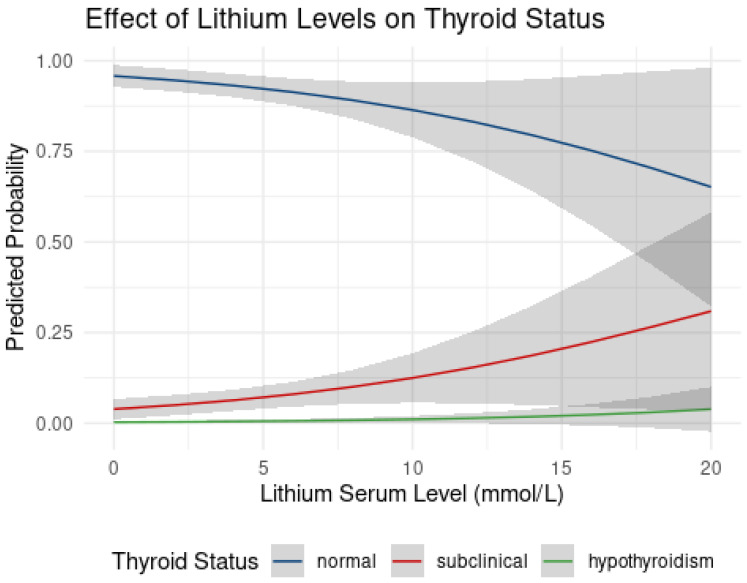
Thyroid effect of lithium levels on predicted thyroid status. Predicted probabilities (through the CLMM model) of being in each thyroid status category (normal, subclinical, hypothyroidism) as a function of lithium serum levels (mmol/L) in patients undergoing lithium therapy. As lithium levels increase, the probability of remaining in the normal thyroid function category (blue) decreases, while the probability of being classified as subclinical (red) or hypothyroid (green) increases. The plot highlights a strong dose-dependent relationship, where higher lithium levels are associated with a greater likelihood of thyroid dysfunction, particularly shifting from normal to subclinical hypothyroidism.

The results indicate that lithium oral daily dose has a significant impact on the progression of thyroid dysfunction, with an odds ratio (OR) of 1.27 (95% CI: 1.11 to 1.45) for each increase of 100 mg/day in the lithium dose (Figure 3). This suggests that for every 100 mg/day increase in lithium dose, the odds of progressing to a more severe category of thyroid dysfunction increase by about 27%.

**Figure 4:**
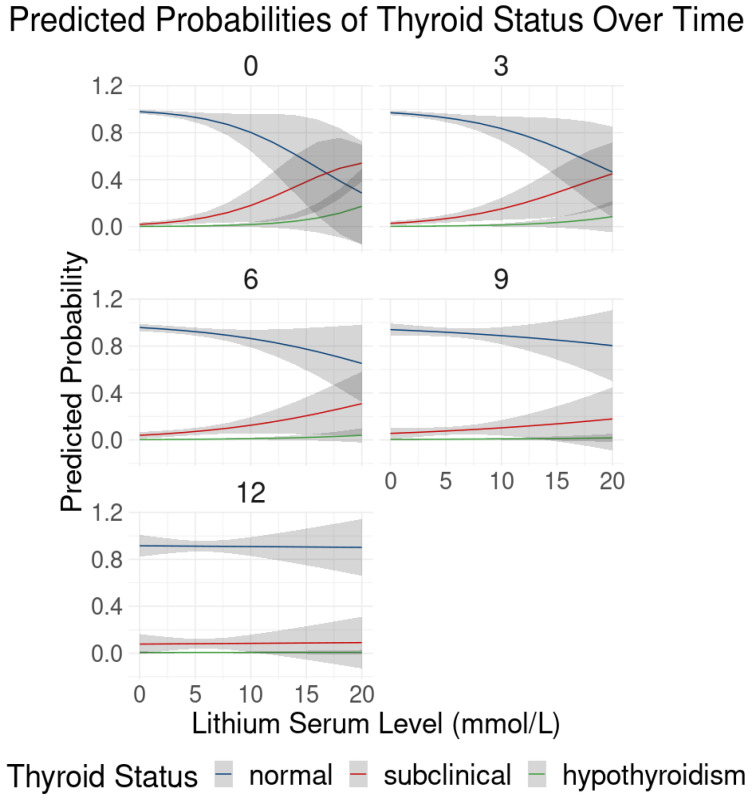
Predicted probabilities of thyroid status over time as a function of lithium levels. This series of line plots shows the predicted probabilities of being in each thyroid status category (normal, subclinical, hypothyroidism) across different time points (0, 3, 6, 9, and 12 months) as a function of lithium serum levels (mmol/L) in patients undergoing lithium therapy. Each panel represents a different time point, illustrating how the relationship between lithium levels and thyroid status evolves over time. At the beginning of treatment (month 0), higher lithium levels are associated with an increased probability of subclinical hypothyroidism and, to a lesser extent, hypothyroidism, while the probability of normal thyroid function decreases. This pattern becomes more pronounced at months 3 and 6, where the likelihood of thyroid dysfunction increases significantly with rising lithium levels. By months 9 and 12, the probabilities stabilize, indicating a potential adaptation or stabilization in thyroid function despite continued lithium exposure.

The interaction between lithium dose and time shows an OR of 0.96 (95% CI: 0.93 to 0.99) per month, suggesting that the impact of lithium dose on thyroid dysfunction decreases slightly over time, indicating a potential adaptation or stabilization of thyroid function with prolonged exposure to lithium (Figure 4).

The random effects component of the model, represented by patient-specific intercepts (variance = 1.903, standard deviation = 1.379), indicates considerable between-subject variability in baseline thyroid function, emphasizing the importance of accounting for individual differences in clinical analyses.

Both models investigated the relationship between lithium treatment and thyroid dysfunction in bipolar patients, with one model using lithium serum levels and the other using lithium oral daily dose as the main exposure variable. Despite differing in the specific lithium measurement, both models produced remarkably similar findings. In each case, lithium exposure significantly increased the odds of progressing to a more severe category of thyroid dysfunction, suggesting a consistent relationship between lithium treatment and thyroid impact. The effect of lithium diminished over time in both models, indicating a potential adaptation of thyroid function with prolonged exposure. Interestingly, the selection process for both models identified the same confounding variables—the baseline TSH, gender, treatment with gabapentin or pregabalin, other antidepressants, and age at onset—demonstrating the robustness of these factors in adjusting for potential confounding effects. Overall, both models highlight the significant influence of lithium treatment on thyroid health, regardless of whether exposure is measured via serum levels or oral dose, while also emphasizing that individual variability plays a substantial role in determining the progression of thyroid dysfunction. The results above are summarized in Supplementary Materials, Table S4.

In addition to the traditional model estimates, we conducted bootstrap analysis with 1000 replications to assess the stability and robustness of the results. The bootstrap approach provided additional confidence in the model’s findings [33].

For the model using lithium serum levels, the bootstrap estimate for the effect of lithium on thyroid dysfunction was 0.290, with a 95% confidence interval ranging from 0.063 to 0.367 using the bias-corrected accelerated (BCa) method [34], and from 0.140 to 0.453 using the percentile method. These intervals largely confirmed the original model’s findings, indicating that the effect of lithium serum levels on thyroid dysfunction is robust.

Similarly, for the model using lithium oral dose as the main exposure variable, the bootstrap estimate was 0.260, with a 95% confidence interval of 0.073 to 0.354 using the BCa method, and from 0.120 to 0.213 using the percentile method. Again, the results were consistent with the original model estimates, reinforcing the relationship between lithium dose and thyroid dysfunction.

These additional analyses indicate that the observed effects of lithium serum levels and oral dose on thyroid status are stable and reliable, further supporting the validity of the study’s findings. The bootstrap distributions, estimates, and confidence intervals for both models are visualized in Supplementary Materials, Figure S2a,b.

## 3. Discussion

In our sample of bipolar patients treated with lithium, we observed notable thyroid function abnormalities. Specifically, 36.7% of patients exhibited alterations in thyroid function, yet only 8.7% required thyroid replacement therapy. This intervention was effective in normalizing TSH and thyroid hormone levels. Among the remaining 28% of patients with abnormal thyroid levels, these abnormalities were transient and normalized over time without the discontinuation of lithium or the need for long-term thyroid replacement therapy. Our analysis, which included the use of an ordinal mixed-effects model, demonstrated that lithium levels significantly influence the risk of developing hypothyroidism, particularly in the early stages of treatment. However, with appropriate management, these thyroid issues did not impede the symptomatic improvement in BD patients.

These findings emphasize the critical importance of continuous monitoring of thyroid function during lithium therapy. Our data indicated statistically significant increases in TSH levels within the first three and six months of treatment, with some patients exhibiting transiently elevated TSH levels. Given these results, it is advisable to adopt a monitoring schedule that includes more frequent checks during the first year of lithium therapy—every three to six months—followed by regular bi-annual assessments. This aligns with the guidelines from the Italian Medicines Agency (AIFA) [35], the National Institute for Health and Care Excellence (NICE) [36], and the U.S. Food and Drug Administration (FDA) [37], which recommend regular monitoring of thyroid function during lithium treatment. Such monitoring protocols should be tailored to individual patient needs, considering other concurrent medical conditions and therapies.

Our study also observed a significant linear decrease in MADRS and MRS scores from the first observation point at three months, indicating clinical improvement in depressive symptoms. Importantly, this improvement occurred regardless of the presence of transient thyroid abnormalities. In cases where severe and symptomatic thyroid dysfunctions arose, they were effectively managed with thyroid replacement therapy. This highlights that with proper management, lithium-induced thyroid dysfunction does not necessarily compromise the overall efficacy of treatment for BD. Clinicians should therefore be vigilant in managing thyroid issues to maintain the continuity and success of lithium therapy.

Previous studies have shown a correlation between thyroid function and treatment response in BD, with lower thyroid function and higher TSH levels associated with longer times to remission and slower response to treatment. Patients with an optimal thyroid profile have been shown to experience better outcomes [38,39,40,41]. For example, Fagiolini and colleagues observed that patients who developed abnormal TSH levels during treatment spent more time in the acute phase of the illness and exhibited higher depressive scores [32]. These findings are consistent with the overlap between clinical manifestations of thyroid dysfunction and depressive symptoms. In our study, the temporary nature of hypothyroidism or the prompt treatment of hypothyroidism, enabled by close monitoring, likely mitigated the impact of thyroid dysfunction on depressive symptoms. However, while there is considerable literature on the impact of hypothyroidism on depressive symptoms in major depressive disorder (MDD), evidence regarding its influence on bipolar depression remains limited.

Our study aligns with the broader literature that demonstrates a link between lithium therapy and thyroid function alterations [26,42,43]. Meanwhile, another study observed that up to 47% of patients developed subclinical hypothyroidism within the first two years of lithium treatment, with a smaller percentage progressing to overt hypothyroidism [21]. Additionally, another study conducted over 15 months showed that the prevalence of clinical hypothyroidism during lithium treatment was 10.4% [44]. Additionally, a previously mentioned study showed a prevalence of abnormal FTI and TSH levels in 38% of bipolar patients treated with lithium [32].

In our study, we found that 8.67% of patients undergoing lithium treatment developed clinical hypothyroidism. This finding aligns with the broader literature, including a study that reported that over 20% of patients developed hypothyroidism within the first year of lithium treatment [45]. Additionally, a comprehensive meta-analysis indicated that lithium treatment significantly increases the odds of developing clinical hypothyroidism, with an odds ratio of 5.78 compared to non-users [46]. Regarding patients who developed subclinical hypothyroidism, as indicated by elevated TSH levels along with normal T3 and T4 values, but which resolved without the need for replacement therapy, none of the patients in our cohort reported any symptoms of hypothyroidism. Nevertheless, it is important to highlight that even short-term hypothyroidism might lead to fatigue, weight gain, depression, cognitive impairment, and cold intolerance, which can impair daily functioning and overall well-being [47]. Therefore, it is crucial to track and monitor even subclinical and/or transient hypothyroidism to ensure it does not negatively impact the patient’s quality of life.

Lithium is known to concentrate in the thyroid at higher levels than in the plasma, potentially inhibiting thyroid hormone release and affecting iodide transport, deiodinase activity, and TSH action [48,49]. These effects contribute to goiter, hypothyroidism, and increased thyroid autoimmunity, particularly in patients with pre-existing conditions. However, it is also important to consider that bipolar disorder itself has been associated with thyroid function alterations. Some studies suggest that patients with bipolar disorder are more likely to have thyroid dysfunction compared to the general population, especially in those with refractory and rapid-cycling forms of the disorder [50]. Disruptions in the hypothalamo–pituitary–thyroid (HPT) axis, which plays a critical role in mood regulation, may significantly impact the clinical course of bipolar disorder. Both hyperthyroidism and hypothyroidism are linked to mood disturbances, with hyperthyroidism often associated with anxiety and mood lability, and hypothyroidism with depressive symptoms [51].

Additionally, the potential impact of other medications taken by our patients as part of their therapy must be considered, as these can also affect thyroid function in clinical practice [42].

In conclusion, our study found that transient thyroid abnormalities in most patients normalized over time without the need to discontinue lithium or initiate long-term thyroid replacement therapy. This finding is crucial for the continued prescribability of lithium, a cornerstone in the treatment of bipolar disorder known for its effectiveness across all phases and its anti-suicidal properties. Despite its narrow therapeutic index and potential side effects, including thyroid dysfunction, lithium remains a vital treatment option. Our findings reinforce the importance of careful monitoring and management of thyroid function during lithium therapy, enabling many patients to continue benefiting from this essential treatment. Clinicians should be well prepared to address and manage potential side effects, such as hypothyroidism, to ensure the continued effectiveness and tolerability of lithium in treating bipolar disorder. Our findings underscore the critical importance of vigilant monitoring and management of thyroid function during lithium therapy. In our study, we found that a well-balanced approach, involving thyroid function tests every three months during the first year, was effective in enabling many patients to continue benefiting from this essential treatment without significant thyroid-related complications. Clinicians should be well prepared to address and manage potential side effects, such as hypothyroidism, to ensure the continued effectiveness and tolerability of lithium in treating bipolar disorder. It is important to note that early diagnosis of lithium-induced hypothyroidism, followed by prompt treatment with thyroid replacement therapy, can allow lithium to remain effective in bipolar patients, as demonstrated in our sample.

## 4. Materials and Methods

### 4.1. Study Design

A retrospective monocentric observational study of patients with bipolar disorder treated with lithium was conducted at the Department of Psychiatry of the University Hospital of Siena at the Santa Maria alle Scotte Hospital in Siena (Ethics Committee approval number: 26967, date of approval: 15 July 2024). A signed informed consent form was obtained from each patient. This article has been prepared in alignment with the guidelines outlined in the ‘Strengthening the Reporting of Observational Studies in Epidemiology’ (STROBE) Statement, specifically following the cohort study checklist [52].

### 4.2. Setting

**Figure 5:**
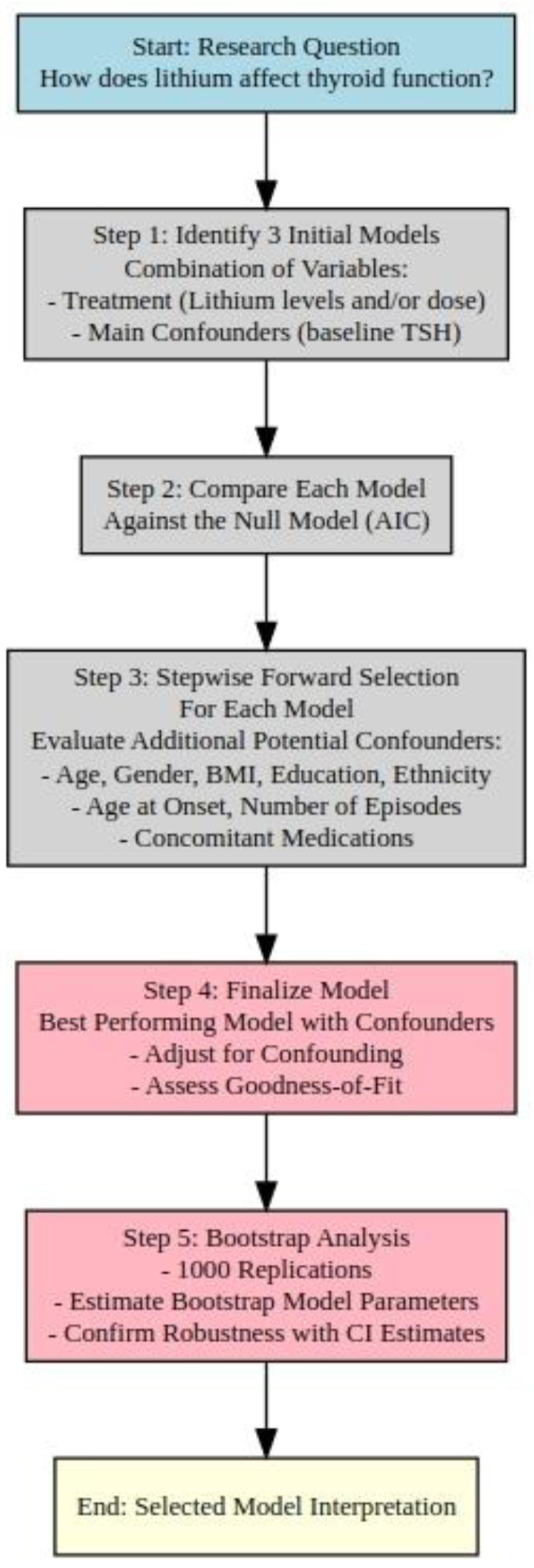
Flowchart of the model selection process for evaluating the impact of lithium on thyroid function. The process involves identifying initial models, comparing against the null model, stepwise forward selection of potential confounders, and finalizing the model based on the best fit using the Akaike Information Criterion (AIC).

The observation period was from January 2017 to May 2024. Patients’ data were collected retrospectively by accessing the medical records of patients consecutively referred to the Department of Psychiatry with a diagnosis of bipolar disorder and initiated on lithium therapy. The data for each patient were collected for a period of 12 months from the initiation of lithium treatment (Figure 5).

### 4.3. Patients’ Inclusion/Exclusion Criteria

Patients were included in the study based on the following inclusion criteria: (1) age ≥ 18 years without distinction of gender and ethnicity; (2) patients diagnosed with bipolar disorder according to DSM5-TR and confirmed by clinical evaluation and treated with lithium; (3) signing of informed consent.

Patients who were unable or unwilling to process data, diagnosed with Alzheimer’s disease, other forms of dementia, cognitive impairment, moderate to severe intellectual disability, or other forms of marked cognitive impairment, had organic brain pathologies or internal pathologies that are not adequately controlled, and pregnant or breastfeeding women were excluded from the study.

### 4.4. Outcomes

The severity of depressive symptoms was assessed according to the guidelines for good clinical practice (Ministerial Decree of 27 April 1992) using the following psychometric scales: the Clinical Impression Global Scale (CGI), Montgomery–Asberg Depression Rating Scale (MADRS), and Mania Rating Scale (MRS), which were used in current clinical practice at day 0, month 3, month 6, month 9, and month 12 of lithium treatment. Thyroid function was assessed by blood chemistry tests with the determination of TSH, T3, and T4 levels at day 0, month 3, month 6, month 9, and month 12, respectively. There were no missing data for any of these variables, and consequently, no methods of data imputation were applied before modeling the thyroid status over time. The tolerability and the presence of any side effects to medications or symptoms of hypothyroidism, such as fatigue, weight gain, and cold intolerance, were assessed at each time point during the patient’s clinical visit.

### 4.5. Statistical Analysis

Descriptive statistics for dichotomous and ordinal variables are given as counts and percentages, while those for quantitative variables are given as the mean, standard deviation (SD), minimum, first quartile, median, third quartile, and maximum. Changes from baseline in thyroid status, categorized as normal, subclinical, or hypothyroidism based on TSH and FT4 levels, were analyzed using a cumulative link mixed-effects model (CLMM).

The cumulative link mixed-effects model (CLMM) provides a detailed analysis of how lithium treatment affects thyroid function over time in patients, considering individual variability. The model uses an ordinal outcome variable that classifies patients into categories of thyroid function: normal, subclinical, and hypothyroidism, based on TSH and T4 levels. This allows the assessment of changes in the probability of being in a higher thyroid dysfunction category over time and with varying lithium levels. This approach accounted for individual variability by including patients as random effects and examined the impact of time (at 3, 6, 9, and 12 months) and lithium serum levels on thyroid status.

According to the approach presented in *Applied Longitudinal Analysis* by Fitzmaurice et al., the ordinal random effects model can be written as follows:

$$log\left(\frac{P\left(Y_{ij}\leq k\vee b_{i}\right)}{1-P\left(Y_{ij}\leq k\vee b_{i}\right)}\right)=\eta_{ij}^{\left(k\right)}=\tau_{k}-\left({X_{ij}}^{\prime }\beta +{Z_{ij}}^{\prime }b_{i}\right)$$

where

- $Y_{ij}$ represents the ordinal thyroid status (normal, subclinical hypothyroidism, clinical hypothyroidism) for patient i at observation j;
- k indexes the ordinal categories with k=1,2, corresponding to the cumulative logit model (i.e., log odds for each threshold);
- $P\left(Y_{ij}\leq k\vee b_{i}\right)$: the cumulative probability of being in category (k) or below, conditional on random effects;
- $\eta_{ij}^{\left(k\right)}$ is the linear predictor for the k-th cumulative logit;
- $\tau_{k}$ represents the threshold (cut-off) parameter for the k-th category;
- ${X_{ij}}^{\prime }\beta$ represents the fixed effects part of the model:

$${X_{ij}}^{\prime }\beta =\beta_{1}X_{1ij}+\beta_{2}X_{2ij}+\beta_{3}X_{3ij}+\beta_{4}X_{4i}+\beta_{5}X_{5i}+\beta_{6}X_{6i}+\beta_{7}X_{7i}+\beta_{8}X_{8i}$$

where

○ $X_{1ij}={\mathrm{month}}_{ij}$;
○ $X_{2ij}={\mathrm{Li}}_{ij}$;
○ $X_{3ij}={\mathrm{month}}_{ij}\times {\mathrm{Li}}_{ij}$;
○ $X_{4i}={\mathrm{baseline}\;\mathrm{TSH}}_{i}$;
○ $X_{5i}={\mathrm{Gender}}_{i}$;
○ $X_{6i}={\mathrm{Gabapentin}/\mathrm{Pregabalin}}_{i}$;
○ $X_{7i}={\mathrm{Other}\;\mathrm{Antidepressants}}_{i}$;
○ $X_{8i}={\mathrm{Age}\;\mathrm{of}\;\mathrm{Onset}}_{i}$.

Here, β is the vector of fixed effect coefficients corresponding to each covariate in the model—${Z_{ij}}^{\prime }b_{i}$ represents the random effects part of the model, with

(1) $${Z_{ij}}^{\prime }b_{i}=b_{i}$$

where $b_{i}$ is the random intercept for patient i, which accounts for individual variability across patients and is assumed to follow a normal distribution: $b_{i}∼N\left(0,\sigma_{u}^{2}\right)$.

This formulation captures the hierarchical structure of the data and the ordinal nature of the response, where fixed effects ${X_{ij}}^{\prime }\beta$ and random effects ${Z_{ij}}^{\prime }b_{i}$ are combined in the linear predictor $\eta_{ij}^{\left(k\right)}$ to model the probability of thyroid status categories.

The main research question was to determine if, and to what extent, exposure to lithium increased the risk of hypothyroidism. We began by identifying nine initial models that included combinations of the following variables: (1) lithium treatment (measured by lithium serum levels and/or oral lithium dose, both with their interactions with time); (2) key suspected confounders, such as baseline TSH levels (which were always included).

At step 0, each of the three models was compared with a null model (with no covariates) using the Akaike Information Criterion (AIC). Following this, a stepwise forward selection process was performed for each model, in which potential confounding variables were added one by one. The variables included in this process were the following: age, gender, BMI, education level, ethnicity, age of onset, number of manic episodes, number of depressive episodes, age at first manic episode, age at first depressive episode, use of mood stabilizers, use of first-, second-, and third-generation antipsychotics, use of benzodiazepines, treatment with gabapentin or pregabalin, treatment with SSRIs, treatment with SNRIs, and treatment with other antidepressants.

We utilized a bootstrap method to evaluate the robustness of our model estimates. Bootstrapping is a non-parametric resampling technique that allows for assessing the stability of statistical estimates by repeatedly sampling with replacement from the original dataset. In this study, we generated 1000 bootstrap replications of our dataset to estimate the distribution of the model parameters [34]. For each replication, we refit the model, enabling us to calculate 95% confidence intervals (CIs) for the main exposure variables (lithium serum levels and oral lithium dose) using both the quantile method and the bias-corrected accelerated (BCa) bootstrap technique as defined by Efron [34]. This process allowed us to confirm the reliability of our findings, with the bootstrap estimates providing further validation of the observed effects on hypothyroidism risk. The results from this bootstrap analysis have been incorporated into our final model output tables.

The model selection process is resumed in Figure 4.

Statistical analyses were performed using R version 4.1.2 through RStudio 2023.06.0 IDE on Ubuntu Linux 22.04.4 LTS. The ordinal mixed-effects model was estimated using the clmm function in the R ordinal package.

## 5. Limitations and Future Perspectives

This study on the impact of lithium on thyroid function in patients with bipolar disorder has potential limitations. Firstly, it would be beneficial to better understand the long-term effects of lithium treatment, as it is a fundamental medication for bipolar disorder and is used for extended periods, even during inter-episode phases. Moreover, it would be useful to expand the sample size, both to increase the numbers and to identify potential differences in geographic areas where the incidence of hypothyroidism may differ from that in the study.

A significant limitation is that the patients were also on other medications; however, rather than demonstrating the well-known correlation between lithium and thyroid function alterations, our study aims to show how these alterations can be monitored and managed without compromising symptom improvement.

Another limitation is related to the scope of our inferential research question, which was strictly limited to quantifying the effect of lithium on thyroid status. Our decision to focus only on this aspect was driven by the need to avoid unnecessary multiple testing, which can reduce statistical power and lead to potential bias. While this strategy ensured the validity of our primary results, it limited our ability to explore other potentially significant variables or outcomes beyond the direct impact of lithium on thyroid function.

Future studies could include larger and more diverse populations to validate these findings further, extending the follow-up period beyond 12 months to better observe long-term thyroid function trends and management outcomes.

## Data Availability

The data presented in this study are available on request from the corresponding author due to (privacy and ethical reasons).

## Supplementary Materials

The following supporting information can be downloaded at: https://www.mdpi.com/article/10.3390/ph17111425/s1, Figure S1: Thyroid Status Distribution Over Time in Patients Undergoing Lithium Therapy. Figure S2a: The histogram shows the bootstrap distribution of lithium serum level estimate. Figure S2b: The histogram shows the bootstrap distribution of lithium oral dose estimate. Table S1: Sociodemographic Characteristics of the Sample; Table S2: Descriptive Statistics for Thyroid Hormones, Lithium Levels, and Lithium Dose Over Time. Table S3: Descriptive Statistics by Substitution Therapy Status. Table S4: Summary of Results from Lithium Serum Level and Lithium Dose Models.
