# Supplementary material for: Insights into the Incidence, Course, and Management of Lithium-Induced Hypothyroidism in Real-World Psychiatric Practice in Italy
Source: Pharmaceuticals (Basel). 2024 Oct 24;17(11):1425. doi: 10.3390/ph17111425 (PMC11597692; doi:10.3390/ph17111425)
Supplement: Supplementary file 1 [file pharmaceuticals-17-01425-s001.zip › pharmaceuticals-3193623-supplementary.pdf]

Supplementary Material

Tables

Table S1. Sociodemographic Characteristics of the Sample.

Characteristic	n (%) or Mean ± SD and Median (IQR)
**Sex**	
Female	77 (51.3%)
Male	70 (46.7%)
Non-binary	3 (2%)
**Education Level**	
Elementary	17 (11.3%)
Middle School	61 (40.7%)
High School	49 (32.7%)
University	23 (15.3%)
**Ethnicity**	
Caucasian	127 (84.7%)
African	1 (0.7%)
Asian	1 (0.7%)
**Age (years)**	Mean ± SD: 45.1 ± 16.9
	Median (IQR): 47 (28.2, 58)
**BMI (kg/m^2)**	Mean ± SD: 25.6 ± 4.78
	Median (IQR): 25.0 (22.5, 27.7)
**Age at Onset (years)**	Mean ± SD: 27.7 ± 14.6
	Median (IQR): 22 (17, 35.5)

Table S2. Descriptive Statistics for Thyroid Hormones, Lithium Levels, and Lithium Dose Over Time.

Variable	Time Point	Mean	SD	Min	1st Quartile	Median	3rd Quartile	Max
**TSH**	0	2.09	1.01	0.01	1.33	1.94	2.77	4.8
	3	2.86	1.86	0	1.58	2.51	3.35	10.1
	6	3.35	4.78	0.55	1.76	2.45	3.41	47.8
	9	2.80	2.62	0.02	1.54	2.28	3.28	27.4
	12	2.73	1.96	0	1.50	2.41	3.36	15.8

Variable	Time Point	Mean	SD	Min	1st Quartile	Median	3rd Quartile	Max
**T3**	0	3.08	1.05	1.6	2.6	2.9	3.2	9.5
	3	2.87	0.724	1.3	2.5	2.8	3.18	9.1
	6	2.89	0.508	1.4	2.6	2.8	3.2	5.1
	9	2.92	0.464	1.9	2.6	2.9	3.2	4.6
	12	3.03	0.932	1.7	2.6	2.9	3.3	12
**T4**	0	11.1	2.60	2.3	9.5	11.0	12.2	22.8
	3	10.4	2.19	2.1	9.3	10.3	11.4	21.7
	6	10.7	2.04	3.1	9.52	10.6	11.9	16.7
	9	10.8	2.13	5.1	9.6	10.6	12.1	21.2
	12	11.0	2.36	4.2	9.5	10.8	12.0	20.1
**Litiemia**	0	0	0	0	0	0	0	0
	3	0.540	0.215	0.2	0.37	0.535	0.67	1.2
	6	0.558	0.264	0.2	0.37	0.52	0.675	1.9
	9	0.544	0.201	0.2	0.392	0.54	0.685	1.15
	12	0.591	0.22	0.2	0.43	0.58	0.745	1.4
**Lithium Dose (100 mg/day)**	0	0	0	0	0	0	0	0
	3	6.82	1.40	6	6	6	7.5	12
	6	6.92	1.50	6	6	6	7.5	12
	9	6.92	1.40	6	6	6	7.5	12
	12	7.10	1.46	6	6	6	9	12

Table S3. Descriptive Statistics by Substitution Therapy Status.

Characteristic	No Substitution Therapy (n = 145)	Substitution Therapy (n = 5)
**Sex**		
Female	73 (50.3%)	4 (80.0%)
Male	72 (49.7%)	1 (20.0%)
**Education Level**		
Elementary	17 (11.7%)	0 (0.0%)
Middle School	57 (39.3%)	4 (80.0%)
High School	49 (33.8%)	0 (0.0%)
University	22 (15.2%)	1 (20.0%)
**Ethnicity**		
Caucasian	123 (84.8%)	4 (80.0%)
African	1 (0.7%)	0 (0.0%)
Asian	1 (0.7%)	0 (0.0%)
**Age (years)**	Mean ± SD: 45.3 ± 17.1	Mean ± SD: 37.6 ± 11.5
	Median (IQR): 48 (28, 58)	Median (IQR): 40 (30, 46)
**BMI (kg/m^2)**	Mean ± SD: 25.6 ± 4.78	Mean ± SD: 27.5 ± 4.92
	Median (IQR): 25.0 (22.5, 27.7)	Median (IQR): 29.1 (22.6, 30.1)
**Age at Onset (years)**	Mean ± SD: 28.0 ± 14.7	Mean ± SD: 18.4 ± 3.51
	Median (IQR): 23 (17, 36)	Median (IQR): 20 (17, 20)
**Number of Manic Episodes**	Mean ± SD: 2.01 ± 1.34	Mean ± SD: 2.20 ± 1.30
	Median (IQR): 2 (1, 3)	Median (IQR): 2 (1, 3)
**Number of Depressive Episodes**	Mean ± SD: 3.39 ± 1.77	Mean ± SD: 3.20 ± 0.837
	Median (IQR): 3 (2, 4)	Median (IQR): 3 (3, 4)
**Age at First Manic Episode**	Mean ± SD: 31.4 ± 15.5	Mean ± SD: 24.2 ± 6.94
	Median (IQR): 25 (19, 45)	Median (IQR): 20 (20, 25)
**Age at First Depressive Episode**	Mean ± SD: 28.7 ± 15.0	Mean ± SD: 18.4 ± 3.51
	Median (IQR): 23 (18, 38)	Median (IQR): 20 (17, 20)
**Use of Mood Stabilizers**	93 (64.1%)	4 (80.0%)
**Use of 1st Gen Antipsychotics**	11 (7.6%)	1 (20.0%)
**Use of 2nd Gen Antipsychotics**	110 (75.9%)	4 (80.0%)
**Use of 3rd Gen Antipsychotics**	77 (53.1%)	3 (60.0%)
**Use of Benzodiazepines**	74 (51.0%)	0 (0.0%)
**Use of Gabapentin or Pregabalin**	53 (36.6%)	1 (20.0%)
**Use of SSRIs**	116 (80.0%)	5 (100.0%)
**Use of SNRIs**	36 (24.8%)	1 (20.0%)
**Use of Other**	73 (50.3%)	2 (40.0%)

Characteristic	No Substitution Therapy (n = 145)	Substitution Therapy (n = 5)
**Antidepressants**		

Table S4. Summary of Results from Lithium Serum Level and Lithium Dose Models

Variable	Model (Serum Levels)	Model (Oral Dose)
**Goodness of Fit (GOF)**		
Log-Likelihood	-301.58	-301.49
AIC	625.16	624.97
**Random Effects (ID)**		
Variance	1.899	1.903
Std. Dev.	1.378	1.379
**Fixed Effects**		
**Month**	OR: 1.13 (95% CI: 1.00 - 1.25)	OR: 1.31 (95% CI: 1.07 - 1.57)
	Estimate: 0.12608 (SE: 0.06376)	Estimate: 0.26757 (SE: 0.10055)
	p = 0.048	p = 0.008
**Lithium**	OR: 1.29 (95% CI: 1.14 - 1.61)	OR: 1.27 (95% CI: 1.11 - 1.45)
(Serum Levels / Dose)	Estimate: 0.25457 (SE: 0.07160)	Estimate: 0.23670 (SE: 0.06799)
	p < 0.001	p < 0.001
**Interaction (Month x Lithium)**	OR: 0.98 (95% CI: 0.96 - 1.01)	OR: 0.96 (95% CI: 0.93 - 0.99)
	Estimate: -0.02049 (SE: 0.01128)	Estimate: -0.03819 (SE: 0.01491)
	p = 0.069	p = 0.010
**Baseline TSH**	OR: 2.41 (95% CI: 1.68 - 3.01)	OR: 2.49 (95% CI: 1.67 - 3.05)
	Estimate: 0.87818 (SE: 0.18487)	Estimate: 0.91251 (SE: 0.18399)
	p < 0.001	p < 0.001
**Sex (Male)**	OR: 0.40 (95% CI: 0.20 - 0.83)	OR: 0.40 (95% CI: 0.20 - 0.83)
	Estimate: -0.91150 (SE: 0.37713)	Estimate: -0.91162 (SE: 0.37677)
	p = 0.016	p = 0.016
**Gabapentin/Pregabalin**	OR: 0.37 (95% CI: 0.17 - 0.81)	OR: 0.38 (95% CI: 0.18 - 0.82)
	Estimate: -0.99376 (SE: 0.41028)	Estimate: -0.97558 (SE: 0.40988)
	p = 0.015	p = 0.017
**Other Antidepressants**	OR: 1.90 (95% CI: 0.90 - 4.02)	OR: 1.88 (95% CI: 0.88 - 4.02)
	Estimate: 0.64345 (SE: 0.38813)	Estimate: 0.63352 (SE: 0.38787)
	p = 0.097	p = 0.102
**Age at Onset**	OR: 0.98 (95% CI: 0.97 - 1.01)	OR: 0.98 (95% CI: 0.97 - 1.01)
	Estimate: -0.02101 (SE: 0.01312)	Estimate: -0.02017 (SE: 0.01310)
	p = 0.109	p = 0.124
**Threshold Coefficients**		
normal->subclinical	Estimate: 4.7019 (SE: 0.8152)	Estimate: 4.9299 (SE: 0.8311)
subclinical->hypothyroidism	Estimate: 7.4184 (SE: 0.8965)	Estimate: 7.6432 (SE: 0.9124)

Figures

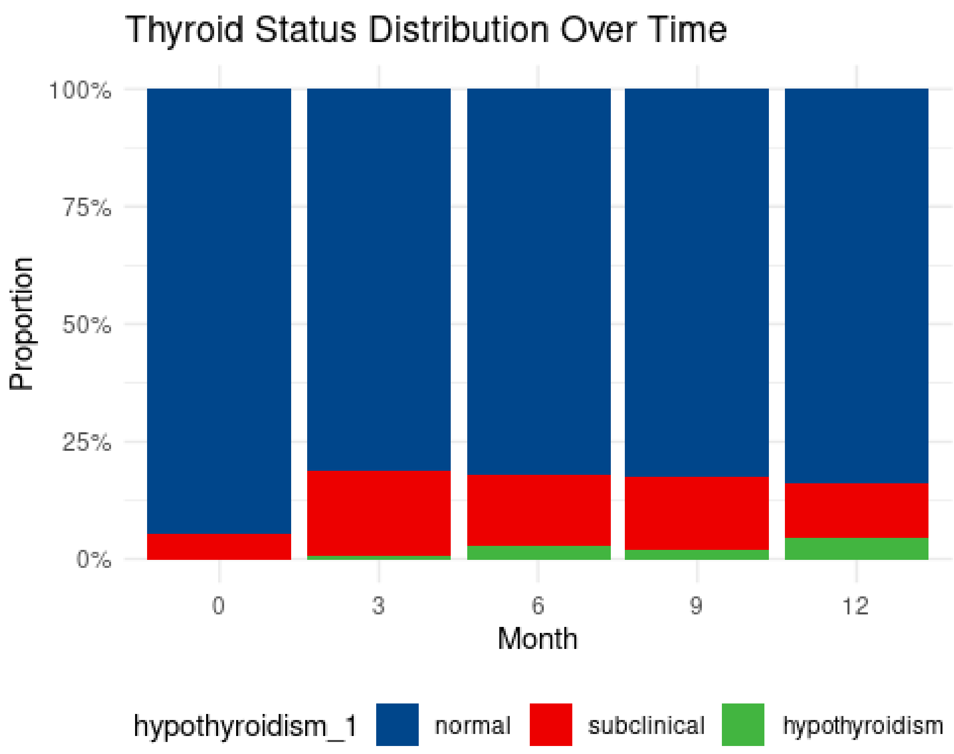

Figure S1. Thyroid Status Distribution Over Time in Patients Undergoing Lithium Therapy. This stacked bar chart shows the distribution of thyroid status categories (normal, subclinical, hypothyroidism) over time in patients treated with lithium. The proportions of patients in each category are displayed for baseline (Month 0) and at 3, 6, 9, and 12 months during the follow-up period. The majority of patients remained in the normal thyroid function category (blue) throughout the observation period, while a smaller proportion developed subclinical hypothyroidism (red), and an even smaller fraction progressed to hypothyroidism (green). The distribution indicates a relatively stable pattern with only slight increases in hypothyroidism and subclinical hypothyroidism over time.

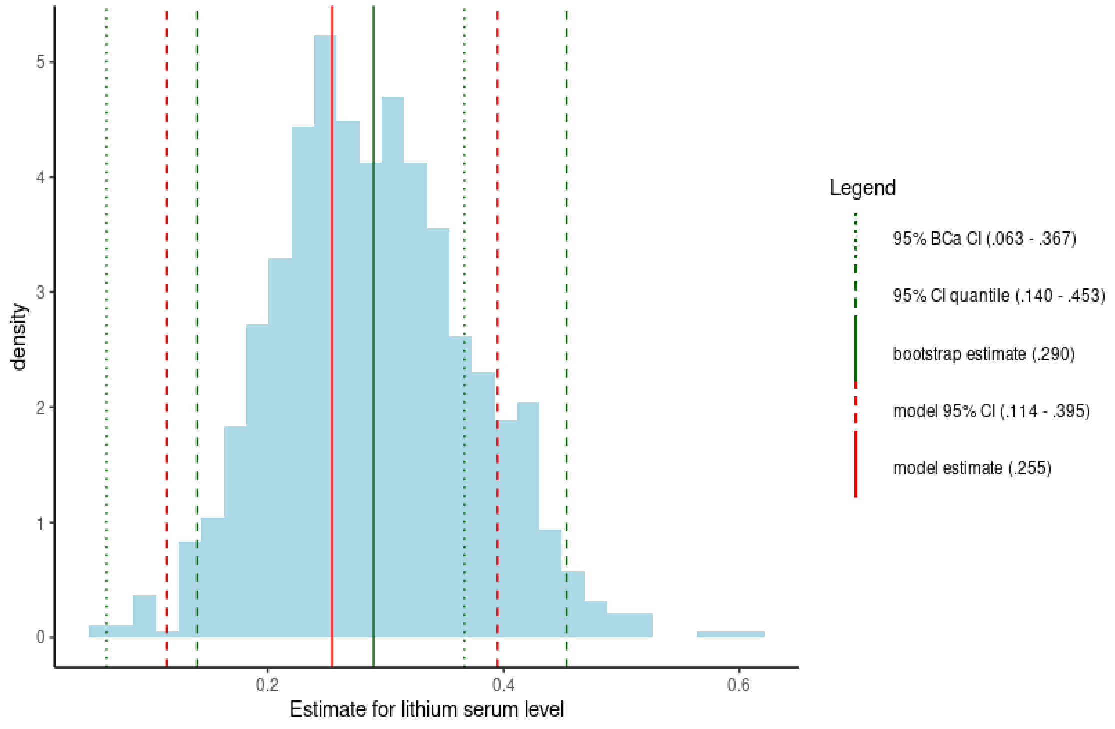

Figure S2a. The histogram shows the bootstrap distribution of lithium serum level estimate. Solid green line represents the mean, dashed green lines the 95% CI quantiles, dotted green lines the bias-corrected and accelerated (BCa) bootstrap CI, and red lines the model estimate with 95% CI.

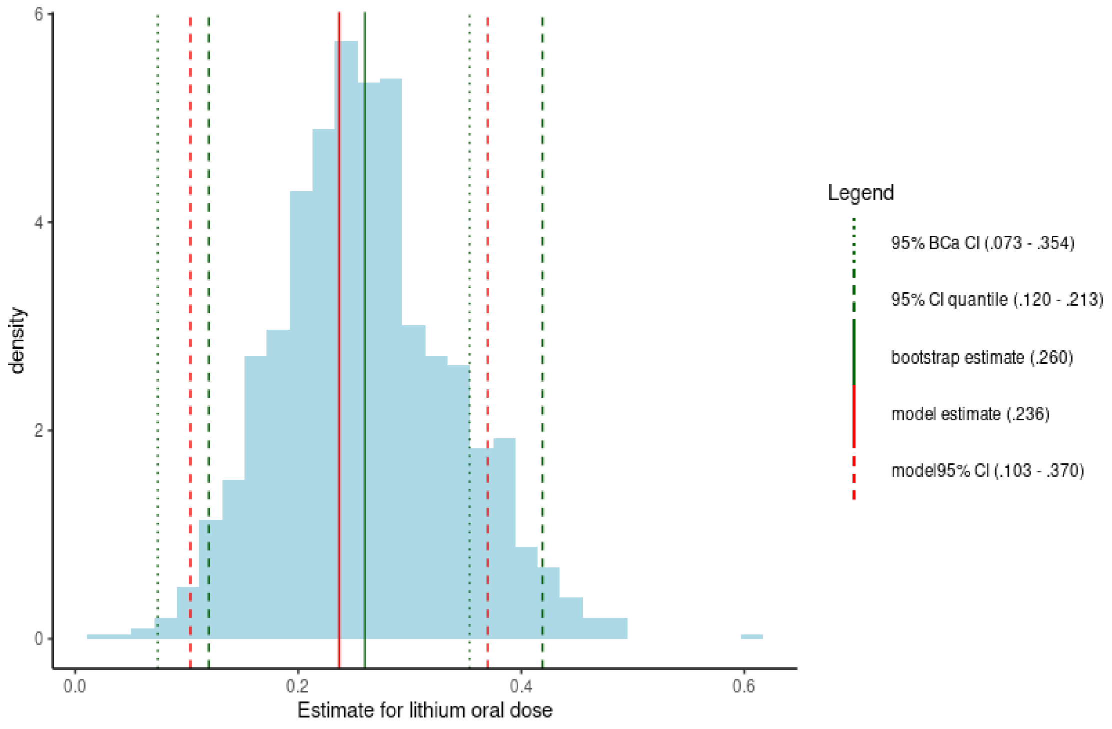

Figure S2b. The histogram shows the bootstrap distribution of lithium oral dose estimate. Solid green line represents the mean, dashed green lines the 95% CI quantiles, dotted green lines the bias-corrected and accelerated (BCa) bootstrap CI, and red lines the model estimate with 95% CI.
